# Identifying Core Genes Related to Low-Temperature Stress Resistance in Quinoa Seedlings Based on WGCNA

**DOI:** 10.3390/ijms25136885

**Published:** 2024-06-23

**Authors:** Lingyuan Zhang, Guofei Jiang, Xuqin Wang, Yutao Bai, Ping Zhang, Junna Liu, Li Li, Liubin Huang, Peng Qin

**Affiliations:** College of Agronomy and Biotechnology, Yunnan Agricultural University, Kunming 650201, China; 2023240197@stu.ynau.edu.cn (L.Z.); 2023210174@stu.ynau.edu.cn (G.J.); 2023240213@stu.ynau.edu.cn (X.W.); 2023210170@stu.ynau.edu.cn (Y.B.); 2021110031@stu.ynau.edu.cn (P.Z.); 2021110026@stu.ynau.edu.cn (J.L.); 2019210130@stu.ynau.edu.cn (L.L.); 2022210173@stu.ynau.edu.cn (L.H.)

**Keywords:** quinoa, low-temperature stress, Weighted Gene Co-Expression Network Analysis, gene interactions networks, TFs

## Abstract

Quinoa is a nutritious crop that is tolerant to extreme environmental conditions; however, low-temperature stress can affect quinoa growth, development, and quality. Considering the lack of molecular research on quinoa seedlings under low-temperature stress, we utilized a Weighted Gene Co-Expression Network Analysis to construct weighted gene co-expression networks associated with physiological indices and metabolites related to low-temperature stress resistance based on transcriptomic data. We screened 11 co-expression modules closely related to low-temperature stress resistance and selected 12 core genes from the two modules that showed the highest associations with the target traits. Following the functional annotation of these genes to determine the key biological processes and metabolic pathways involved in low-temperature stress, we identified four important transcription factors involved in resistance to low-temperature stress: *gene-LOC110731664*, *gene-LOC110736639*, *gene-LOC110684437*, and *gene-LOC110720903*. These results provide insights into the molecular genetic mechanism of quinoa under low-temperature stress and can be used to breed lines with tolerance to low-temperature stress.

## 1. Introduction

In plants, low-temperature stress, which can be categorized as either freezing or chilling stress depending on the temperature, is a serious abiotic stress. Chilling can lead to a decline in absorptive function, decreased photosynthesis, a disruption of the formation layer, and toxicity from hydrogen peroxide accumulation in the body of the plant [1], whereas freezing may lead to a number of negative impacts, such as wilting, dwarfing, and chlorosis [2]. The cold tolerance mechanism of plants can be categorized into cold or freezing tolerance [3]: cold tolerance reflects the ability of plants to respond favorably to growing conditions in the temperature range of 0–15 °C, that is, to survive and not exhibit limited growth [4,5], whereas freezing tolerance reflects a plant’s ability to respond favorably to subzero temperatures [6]. When exposed to low temperatures, plants alter their gene expression patterns and protein products to maximize cold tolerance mechanisms [7,8], which are determined by crop–environment–symbiosis interactions [9]. At low temperatures, plants sense low-temperature signals and ameliorate the adverse effects on their growth and development by altering gene expression, producing antioxidant enzymes, and changing membrane composition, triggering strains and downstream signaling chains to induce appropriate defense mechanisms that improve low-temperature stress tolerance [10,11,12]. AP2/ERF, MYB, NAC, and bHLH are a family of transcription factors (TFs) found in different plant species that are capable of cold-stress perception, signaling, and transcriptional regulation [13,14]; when the corresponding signaling pathway is activated, these TFs can systematically regulate the promoter region of genes by binding to the cis-acting elements of gene expression to improve plant cold tolerance [15]. However, the selection of suitable stress markers is also required when studying cold tolerance in plants. Yu et al. [16] demonstrated that proline and malondialdehyde were suitable low-temperature stress markers when studying transcription factors for cold tolerance in sweet potato; similarly, Wang et al. [17] used proteins, soluble sugars, proline, malondialdehyde, and peroxidase as low-temperature stress markers. This shows that appropriate protein markers and dyes are suitable stress markers.

Quinoa (*Chenopodium quinoa* Wild.) is an annual broadleaf plant, known as a pseudocereal, which has gained attention as a whole nutrient crop. The seeds contain abundant protein, a wide range of minerals and polyphenols, and other beneficial components [18,19]. Moreover, quinoa contains 16 amino acids, including 9 essential amino acids, with greater abundance of lysine, histidine, and methionine than cereal proteins [20]. In addition to nutritional richness, quinoa is important for its resistance to abiotic stresses, exhibiting tolerance to adverse conditions such as drought and low and high temperatures [21,22]. Some quinoa varieties are even tolerant of a certain degree of frost [23] and are typically less affected by frost than most crops. Furthermore, different cultivars and growth periods correspond to different sensitivities to low-temperature stress, with lines containing high levels of soluble sugars exhibiting greater tolerance to low temperatures [24]. 

The Weighted Gene Co-Expression Network Analysis (WGCNA), which is used to screen for modules containing multiple highly correlated genes, employs a signature gene network approach to correlate the screened gene modules with each other and with relevant sample traits, which can then be further mined to identify core genes related to those traits [25]. Currently, WGCNA the analysis is widely used in the field of bioinformatics. For example, Shen et al. used WGCNA to analyze the specific modules and candidate genes that are significantly related to the metal ion content of melon [26]. In addition, Wang et al. used WGCNA to analyze five developmental stages of two peach species and identified three candidate genes related to malate and one to citrate content, which contained three genes encoding the WRKY50, MYB62, and bHLH1 TF families [27]. Interestingly, Zhang et al. used WGCNA to identify 15 core genes related to proanthocyanidin accumulation in the fruits of *Salvia divinorum* and then obtained 10 highly related candidate genes [28]. Similar to their study, Wang et al. sequenced the developmental-stage transcriptomes of fruits from three jujube varieties and identified nine structural genes and 49 regulatory genes that are highly correlated with proanthocyanidin accumulation, of which 16 TFs (MYB, ERF, and NAC) were deemed hub genes [29]. However, few studies have performed molecular-level analyses of quinoa seedlings under low-temperature stress.

Therefore, in this study, we employed two independently selected quinoa lines as test materials [30] and then used WGCNA to construct gene co-expression networks under three environmental conditions: freezing, chilling, and ambient temperatures. This research elucidates the molecular genetic mechanism of quinoa, as well as that of other crops with similar physiological mechanisms, under low-temperature stress. 

## 2. Results

### 2.1. Physiological Changes in Quinoa Seedlings under Low-Temperature Stress

**Figure 1:**
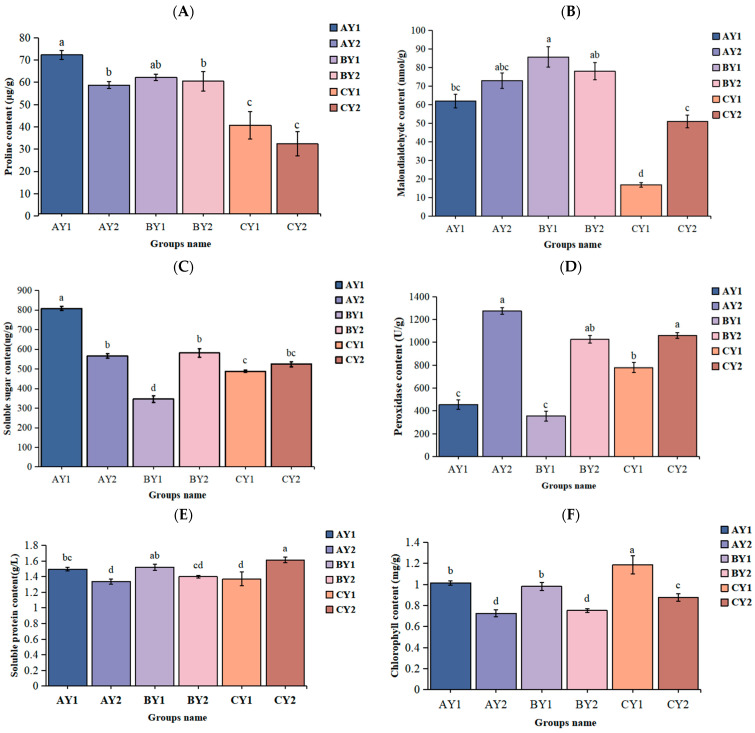
Physiological changes in quinoa seedlings under different stresses: (**A**) proline content, (**B**) malondialdehyde content, (**C**) soluble sugar content, (**D**) peroxidase content, (**E**) soluble protein content, and (**F**) total chlorophyll content. Identical letters (a–d) indicate no significant differences (*p* > 0.05), and groups with different letters indicate significant differences (*p* < 0.05).

Under low-temperature stress, the proline content of both sensitive and resistant lines was significantly lower in the control than in the treatment groups. The proline content was higher in the sensitive variety than in the tolerant variety, and it was significantly higher in AY1 than in AY2 (Figure 1A). The malondialdehyde content of both varieties was significantly higher in the treatment groups than in the control (Figure 1B). The soluble sugar content of the two varieties did not change significantly at ambient temperature but was significantly higher in the tolerant variety than in the sensitive variety at 5 °C, and several stress treatments were significantly higher than the control at −2 °C and 5 °C, except for BY1 (Figure 1C). The peroxidase content was significantly lower in the sensitive variety than in the low-temperature-tolerant variety (Figure 1D). The soluble protein content was significantly lower in the sensitive control than in the two treatments, significantly higher in the tolerant control than in the two treatments, and significantly higher in the sensitive variety than in the tolerant variety for both treatments (Figure 1E). Finally, the total chlorophyll content was significantly higher in the control than in the treatment groups for both lines (Figure 1F). 

Regarding the physiological changes in quinoa seedlings under different stresses, we found that six physiological indicators were correlated at low temperatures (Table 1). Proline was negatively correlated with four of the remaining five indicators, but only the correlation with malondialdehyde was significant. Similarly, chlorophyll was negatively correlated with four indicators, but only the correlation coefficients with malondialdehyde and peroxidase were highly significant. 

### 2.2. Construction of Gene Co-Expression Network

**Figure 2:**
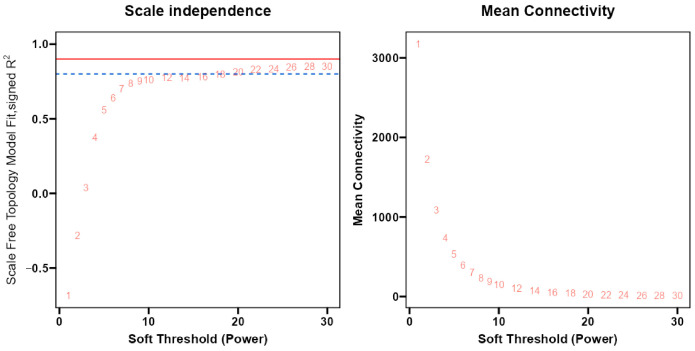
Scale-free fitting indices for soft threshold power and average connectivity. X-axis in both plots represents the weight parameter β. Y-axis in the left plot represents the correspondence between β and the goodness-of-fit (R^2^) for neighbor-joining matrices after transforming the scale-free network assumptions. The red line is the standard line for optimal threshold (β-value) selection; the blue line is the reference line when the β-value is not reached. R^2^ > 0. 8 when the soft threshold value is 22. Y-axis in the right plot represents the average neighbor-joining functions of all genes among the corresponding gene modules, as determined by the representation of the corresponding β-value-transformed neighbor-joining matrices.

After filtering the low-expression genes, the clustering analysis based on gene expression indicated no outlier samples (Supplementary Figure S1). To select the appropriate weighting coefficient, we used the pickSoftThreshold function to calculate the optimal value for the fitting index (R^2^) of the scale-free network as 0.81 when the soft threshold was 22 (Figure 2). 

**Figure 3:**
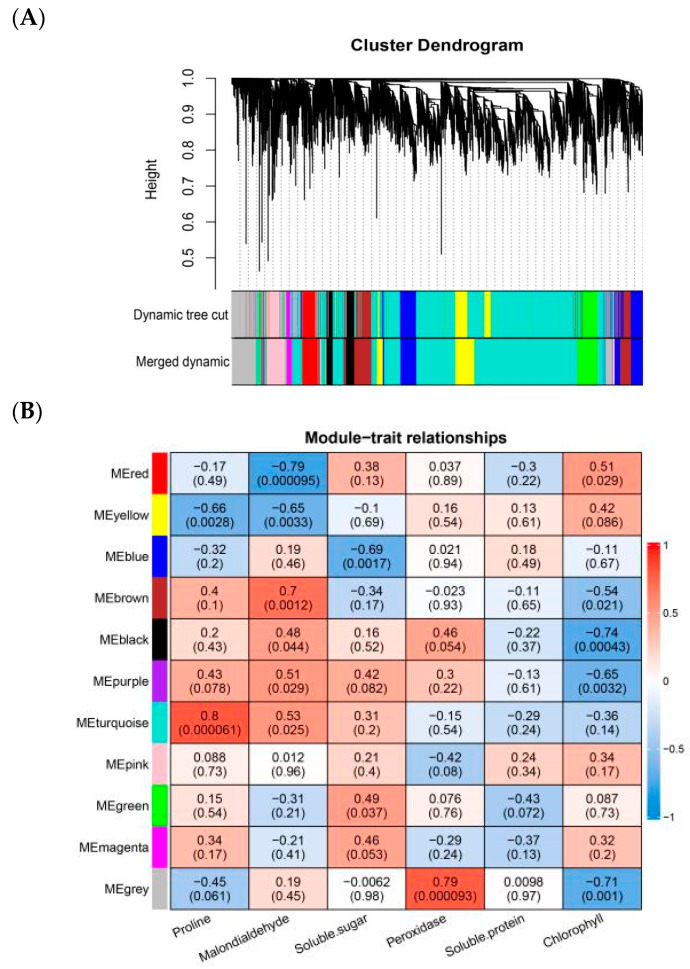

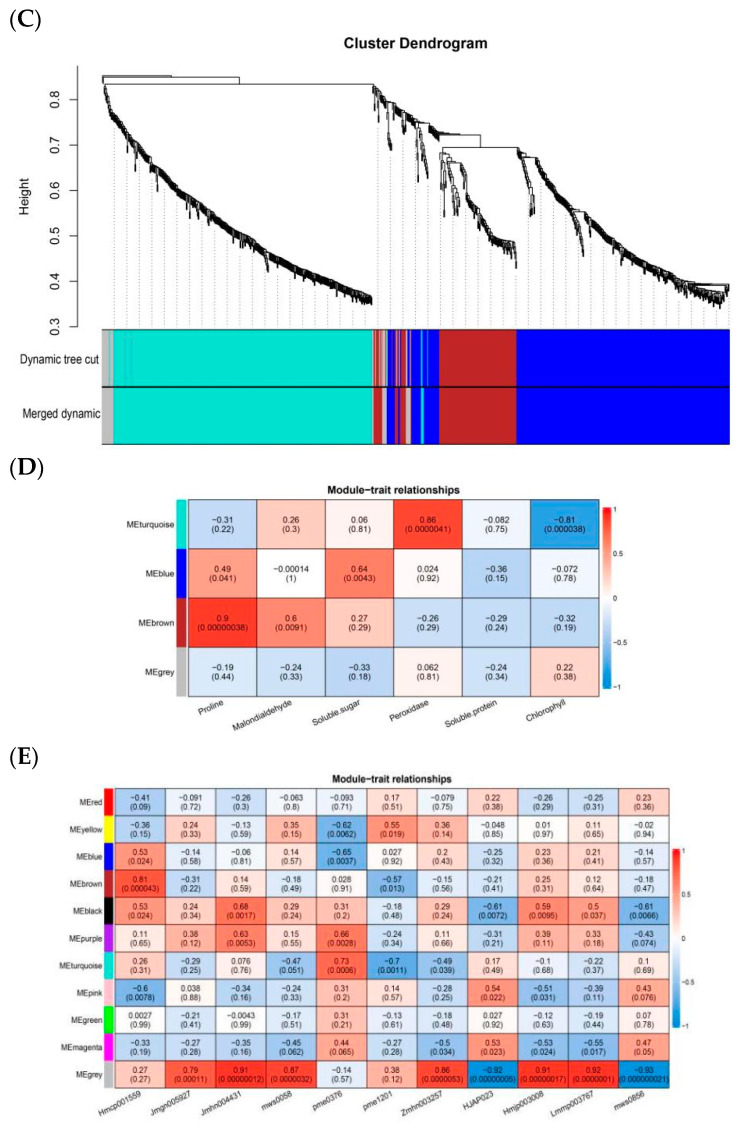
Weighted Gene Co-Expression Network Analysis diagram. (**A**) Gene Cluster Tree. (**B**) Heatmap of gene module correlations with physiological traits. (**C**) Metabolite Cluster Tree. (**D**) Heatmap of metabolite module correlations with physiological correlations. (**E**) Heatmap of gene module correlations with metabolites. The y-axis shows the module name, with each row representing a module, and the x-axis shows the trait name, with each column representing a trait. The numbers in the rectangular boxes indicate the correlation coefficients and corresponding *p*-values between physiological traits and modules. The strength and direction of the correlations are shown on the right side of the heat map (where blue indicates negative correlations, and red indicates positive correlations).

A dynamic hybrid cropping method was used to divide the co-expression modules. After merging the small modules with high similarity, we obtained 11 modules (Figure 3A), each corresponding to a color, with the highest number of genes in the turquoise module (3856), followed by the gray module (1005). Physiological traits were then correlated with the gene modules (Figure 3B). The turquoise module had the most significant positive correlation with proline (r = 0.8, *p* = 0.000061), the gray module had a positive correlation with peroxidase (r = 0.79, *p* = 0.000093) and chlorophyll (r = −0.71, *p* = 0.001), and the red module was negatively correlated with malondialdehyde (r = −0.79, *p* = 0.000095). 

According to the correlations between physiological data and metabolites (Supplementary Table S3), we constructed four modules using WGCNA (Figure 3C). The brown module had the most significant correlation with proline (r = 0.9, *p* = 0.00000038), followed by the turquoise module with peroxidase (r = 0.86, *p* = 0.0000041) and chlorophyll (r = −0.81, *p* = 0.000038) (Figure 3D). A variety of flavonoids and alkaloids were mined in the brown, turquoise, and gray metabolite modules (Supplementary Table S4), which coincided with the differential metabolites that were highly expressed (Supplementary Table S5). The differential metabolites were then associated with the genes as trait data (Figure 3E). The results showed that the gray module was strongly positively correlated with most of the flavonoid differential metabolites. 

### 2.3. GO and KEGG Enrichment Analysis of Relevant Specificity Modules

**Figure 4:**
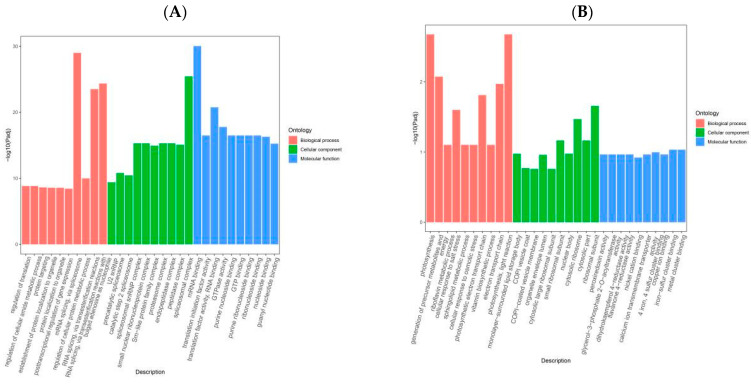

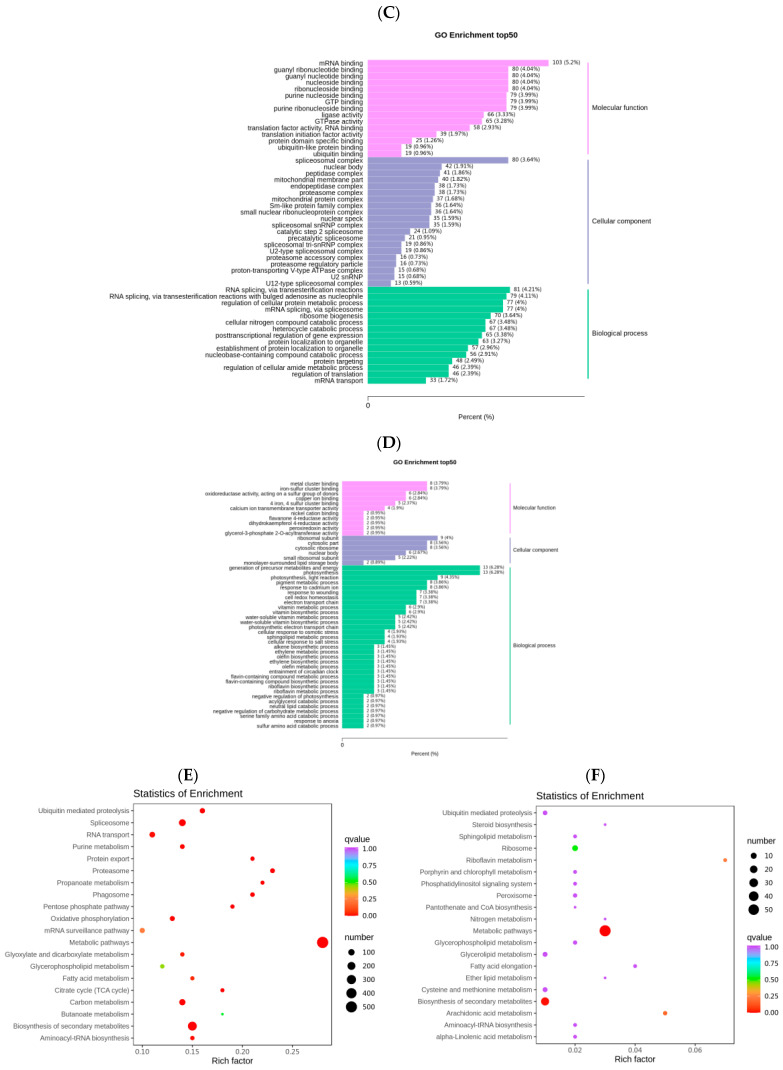
The turquoise gene modules are (**A**,**C**,**E**), and the gray gene modules are (**B**,**D**,**F**), where (**A**,**B**) are GO annotation analyses, (**C**,**D**) are GO enrichment analyses, and (**E**,**F**) are KEGG enrichment analyses. The vertical coordinates of the GO annotation analyses represent the number of genes associated with the GO terms, and the horizontal coordinates represent the GO terms for BP, CC, and MF. The vertical coordinates of the GO enrichment analyses represent the percentage of genes in the GO enrichment terms and the horizontal coordinates represent the percentage of genes in GO terms; the vertical coordinates of the KEGG enrichment analysis represent metabolic pathways, and the horizontal coordinates represent the percentage of genes in KEGG terms.

To explore the functional classification and metabolic pathways of low-temperature stress-responsive genes, Gene Ontology (GO) and Kyoto Encyclopedia of Genes and Genomes (KEGG) were performed on the turquoise and gray modules, respectively (Figure 4). GO pathways can be categorized into biological process (BP), molecular function (MF), and cellular component (CC) pathways. The turquoise module was enriched in the 372 CC, 2177 BP, and 742 MF pathways, but it was predominantly enriched in mRNA binding (GO: 0003729), spliceosome complex (GO: 0005681), and U12-type spliceosome complex (GO: 0005689) (Figure 4C). The gray module was enriched in 764 BP, 145 CC, and 315 MF pathways, but predominantly metal cluster binding (GO: 0051540), iron–sulfur cluster binding (GO: 0051536), ribosomal subunits (GO: 0044391), precursor metabolite production and energy (GO: 0006091), and photosynthesis (GO: 0015979). The GO enrichment analysis showed that the growth and development of quinoa seedlings under low-temperature stress were predominantly affected by influencing enzymatic reactions and metabolic processes (Figure 4D). 

The KEGG enrichment analysis of genes in the two specificity modules showed that genes in both the turquoise and gray modules were significantly enriched in the metabolic (ko01100) and biosynthesis of secondary metabolite (ko01110) pathways (Figure 4E,F). In addition, genes in the turquoise module were enriched in the glyoxylate and dicarboxylate metabolism (ko00630) and pentose phosphate (ko00030) pathways. These results suggest that genes within these modules play key roles in the response of quinoa seedlings to low-temperature stress by regulating the metabolic, secondary metabolite biosynthesis, pentose phosphate, and glyoxylate and dicarboxylic acid metabolism pathways. 

### 2.4. Identification of Core Genes and Construction of Interaction Network

**Figure 5:**
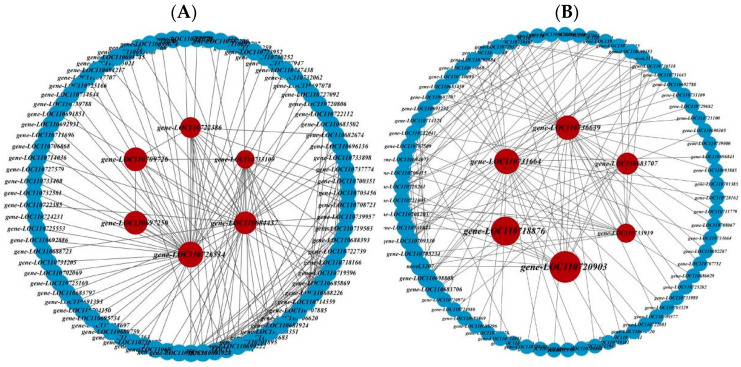
Gene network interaction map. Candidate hub genes for Meturquoise (**A**) and Megray (**B**) from the network analysis of interactions with known core genes. The red color represents the genes screened for the core genes, each node in the network represents a gene, and the edges represent the relationships between the genes.

Considering that the turquoise and gray modules exhibited relatively strong correlations with proline and flavonoids, respectively, and may have potential genes responding to low-temperature stress, these two modules were used to construct gene interaction networks. The genes with the top 20 module eigengene-based connectivity (KME) values in each module were selected as hub genes, and the core genes were screened by calculating the betweenness values using the cytoNCA plug in of cytoscape 3.9.1 software. Ultimately, we obtained 12 core genes (Figure 5). The turquoise module contained six core genes, *gene-LOC110684437*, *gene-LOC110726534*, *gene-LOC110697250*, *gene-LOC110709726*, *gene-LOC110722386*, and *gene-LOC110733109*; and the gray module contained six core genes, *gene-LOC110731664*, *gene-LOC110736639*, *gene-LOC110733919*, *gene-LOC110683707*, *gene-LOC110718876*, and *gene-LOC110720903*. After the promoter analysis of structural genes (Supplementary Table S6), we found that 30 structural genes contained homeopathic elements that bind to the MYB TF family, of which two had MYB binding sites involved in the regulation of flavonoid biosynthesis genes, 15 had MYB binding sites involved in light response, and 13 had MYB binding sites involved in drought induction. 

### 2.5. Transcription Factors under Low-Temperature Stress

**Figure 6:**
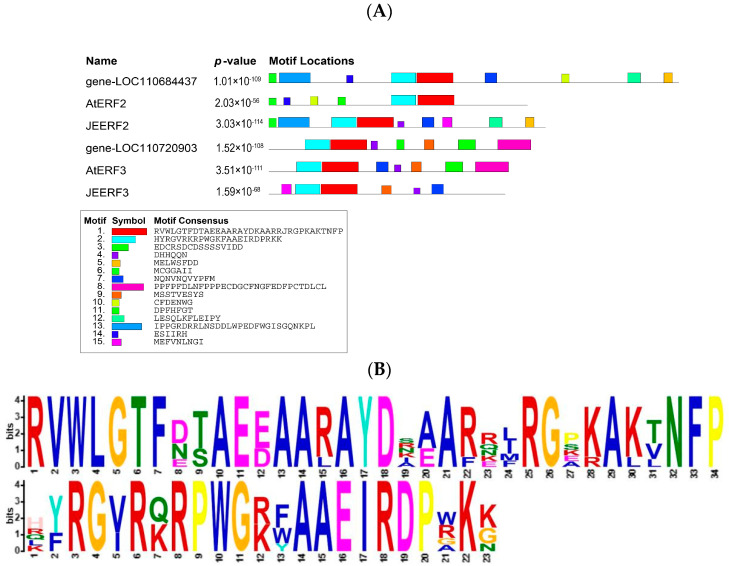
Comparative analysis of AP2/ERF family genes. (**A**) Conserved motif analysis; AtERF is a transcription factor family of Arabidopsis, and JEERF is a transcription factor family of tomato. (**B**) Conserved motif sequence tags.

Five TFs were identified among the 12 core genes (Table 2). *Gene-LOC110684437* and *gene-LOC110720903* belong to the AP2/ERF family of TFs, which show resistance to low-temperature stress in many crops. *Gene-LOC110731664*, *gene-LOC110736639*, and *gene-LOC110733919* belong to MYB-related, NAC, and C2C2-CO-like TF families, respectively. The two genes encoding the AP2/ERF family of TFs (*gene-LOC110684437* and *gene-LOC110720903*) were present in both the turquoise and gray modules and were identified by motifs. Therefore, these two genes with resistance to low-temperature stress were compared to cold-resistance TFs previously identified in Arabidopsis (AtERF2 and AtERF3) and tomato (JEERF2 and JEERF3), respectively (Figure 6A). Fifteen motifs were identified in the six TFs, all of which had at least two identical conserved motifs (Figure 6B). *Gene-LOC110684437* had five and seven identical motifs with AtERF2 and JERF2, respectively. *Gene-LOC110720903* had six and four identical motifs with AtERF3 and JEERF3, respectively. The gene function annotation results in Table 2 showed that both AP2/ERF TFs were ethylene-responsive TFs and may play an important role in the response mechanism of quinoa to low-temperature stress. 

### 2.6. Real-Time Fluorescence Quantitative PCR Validation

**Figure 7:**
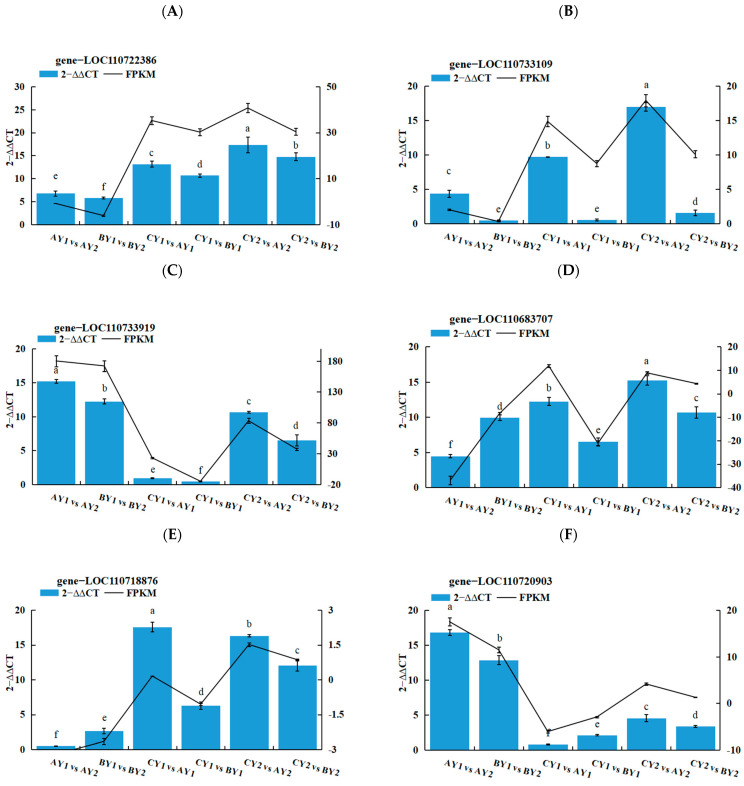
(**A**–**F**) Validation of gene expression levels of randomly selected core genes by RT-qPCR. Identical letters (a–f) indicate no significant differences (*p* > 0.05), and groups with different letters indicate significant differences (*p* < 0.05).

Six genes were selected from the core genes for real-time fluorescence quantitative PCR (RT-qPCR), and three replicates were set up for each reaction. To calculate 2^−ΔΔCT^ and SD, 2^−ΔΔCT^ was used to analyze the normalized expression of each sample, and the FPKM (fragments per kilobase of exon per million fragments mapped) and SD of the validation genes were also calculated. Based on the 2^−ΔΔCT^ of the validation genes and the FPKM of the sequenced genes, the results showed that the expression trends detected by RT-qPCR matched well with the RNAseq data, which proved the reliability of gene expression in this study (Figure 7A–F and Table 3).

## 3. Discussion

Quinoa originates from the Andes, where low temperatures and frequent frosts create the potential for local species to withstand low-temperature stress conditions; however, different varieties and reproductive stages of quinoa exhibit substantial disparity in their tolerance to low temperatures [31,32]. Low temperature not only leads to membrane damage, reduced ATP supply, accumulation of toxic compounds, imbalance of ion supply, and solute leakage, but exposure to low temperatures at the seedling stage can also lead to weak seedlings and, in severe cases, impaired growth and development or even plant death, which affects the quality and yield of quinoa [33,34].

In this study, Dianli 2324 (low-temperature-sensitive variety) and Dianli 281 (low-temperature-tolerant variety) were used to analyze quinoa responses to three different temperature conditions at the molecular level. The proline contents were higher in quinoa seedlings under low-temperature stress, presumably because the seedlings synthesized a large amount of proline in response to low-temperature stress, which agrees with the conclusions of Kaur [35]. Malondialdehyde showed a significant negative correlation with the proline content. Indeed, Uzal et al. inhibited malondialdehyde production through the application of exogenous proline to achieve improved resistance to low-temperature stress for tomato plants [36]. Malondialdehyde is one of the end products of lipid peroxidation by free radicals, whereas proline is a reactive oxygen scavenger that has antioxidant properties and reduces lipid peroxidation [37]. 

According to the WGCNA analysis, proline and peroxidase were strongly positively correlated, and many metabolites with high expression were flavonoids. Flavonoids form non-promotional antioxidant defense systems in plants and have antioxidant properties that scavenge reactive oxygen species to protect plant cells from oxidative damage [38]. According to the GO and KEGG enrichment analysis, differential genes in the turquoise module were mainly enriched in the pentose phosphate pathway and glyoxylate and dicarboxylic acid metabolism. Notably, Sarkar et al. found that regulating the pentose phosphate pathway could induce better cold stress tolerance in cool-season turfgrasses [39], whereas Xu et al. found that winter radish oilseed rape regulates the pentose phosphate and glyoxylate and dicarboxylic acid metabolism pathways to better cope with cold stress [40]. Therefore, our results of differential gene enrichment under low-temperature treatment are consistent with those of previous studies. 

Five TFs were obtained by screening the core genes, *gene-LOC110731664*, *gene-LOC110736639*, and *gene-LOC110733919* belong, to the MYB-related, NAC, and C2C2-CO-like TF families, respectively, and *gene-LOC110684437* and *gene-LOC110720903* belong to the AP2/ERF TF family. By introducing MbMYB108 identified from *Malus baccata* (L.) Borkh into Arabidopsis thaliana, Yao et al. showed that MbMYB108 may play an important role in the response of Arabidopsis thaliana to cold by enhancing the scavenging ability of reactive oxygen species [41]. Moreover, Yao et al. found that introducing MbMYB4 into Arabidopsis thaliana significantly increased the proline and chlorophyll content and significantly decreased the malondialdehyde content of Arabidopsis thaliana, which greatly improved cold tolerance in this crop [42]. Thus, the MYB TF family plays an important role in plant resistance to low-temperature stress. The results of the promoter analyses also indicate that many structural genes have homeotic elements that bind to the MYB TF family, and some of these structural genes have MYB binding sites involved in the regulation of flavonoid biosynthesis genes, indirectly suggesting that flavonoids may be regulated by the MYB family and play an important role in low-temperature stress. In this study, we screened quinoa genes for the MYB TF family *gene-LOC110731664*, which may also play a role in quinoa resistance to low-temperature stress. 

NAC TFs play important roles in resistance to cold stress in a variety of plants. For example, Hou et al. isolated the CaNAC064 gene from chili pepper leaves, and based on the silencing of CaNAC064 in chili pepper plants and overexpression in Arabidopsis, etc., the results showed that CaNAC064 was able to positively regulate the cold tolerance of plants under low-temperature stress [43]; Pang et al. overexpressed the AmNAC11 gene in Arabidopsis, which could significantly enhance the tolerance to freezing stress from the onset [44].Therefore, we suggest that the NAC TF family is important for plant resistance to low-temperature stress, and that *gene-LOC110736639* of the NAC TF family may enhance cold tolerance in quinoa seedlings. 

AP2/ERF TFs are one of the largest plant-specific transcriptional regulators and an important TF family related to cold stress. The ethylene response factor (ERF) subfamily of TFs plays an important role in low-temperature stress in plants. Li et al. overexpressed VvERF63 in *Arabidopsis thaliana*, which resulted in improved cold tolerance in *Arabidopsis thaliana* at the seedling and maturity stages, thus suggesting that VvERF63 is positively involved in cold response [45]; ERF105 is a cold-regulated TF gene in *Arabidopsis thaliana*, and overexpressed plants were more frost-tolerant [46]. Furthermore, Wu et al. found that JERF3 enhanced tolerance to cold stress in tobacco through the transcriptional activation of gene expression, leading to the reduced accumulation of reactive oxygen species [47]. The genes identified in this study as belonging to the AP2/ERF TF family (*gene-LOC110684437* and *gene-LOC110720903*) may play a similarly important role in cold stress response. Moreover, a comparison of these genes with AtERF3, JEERF3, AtERF2, and JEERF2, previously identified as the core TFs of low-temperature stress resistance, showed similarities among the six TF motifs, all of which contained the AP2 domain. Therefore, we speculate that *gene-LOC110684437* and *gene-LOC110720903* help quinoa seedlings resist low-temperature stress. 

## 4. Materials and Methods

### 4.1. Quinoa Planting and Growing Conditions

This study was performed at the Modern Agricultural Education and Research Base of Yunnan Agricultural University in Xundian County, Kunming City, Yunnan Province (102°41′ E, 25°20′ N). Two independently selected lines of low-temperature-sensitive (Dianli 2324) and highly low-temperature-resistant (Dianli 281) quinoa were planted in seedling trays (54 × 28 × 12 cm), with normal field management implemented during the early stage. When the seedlings of the two lines reached the stage of 6–8 true leaves, the seedlings were transferred to either freezing (−2 °C), cold (5 °C), or room-temperature (22 °C) conditions; all other conditions were unchanged. Once irreversible damage was observed in Dianli 2324 seedlings in the −2 °C treatment group, we collected two samples each from the two treatment groups (−2 °C and 5 °C) and the control group (22 °C), with three biological replicates; thus, 18 samples were collected in total. The aboveground part sampling was snap-frozen in liquid nitrogen and stored at −80 °C prior to analysis. Quinoa samples from the −2 °C, 5 °C, and 22 °C groups were named AY1, BY1, and CY1 for Dianli 2324 and AY2, BY2, and CY2 for Dianli 281, respectively. 

### 4.2. Data Acquisition

Quinoa leaves were sent to Wuhan Metwel Biotechnology Co., Ltd. (Wuhan, China, http://www.metware.cn/ (accessed on 6 June 2021)) for transcriptome sequencing and broadly targeted metabolome analysis. Transcriptome data (https://www.ncbi.nlm.nih.gov/genome/?term=quinoa (accessed on 13 June 2024)) were used to prepare samples according to the method of Fan et al. [48]. RNA quantification and characterization were then performed; RNA purity was detected using a NanoPhotometer^®^ spectrophotometer (IMPLEN, Life Technologies, Foster City, CA, USA), and RNA integrity was assessed using the RNA Nano 6000 assay kit for the Bioanalyzer 2100 system (Agilent Technologies, CA, USA), followed by library construction. Subsequently, the insert size of libraries was detected using Agilent 2100, and the effective concentration of the libraries was accurately quantified (>2 nM) by real-time quantitative PCR once the insert size met the expectation. Library inspection was then completed, and the data were analyzed for quality control according to the method of Wang et al. [49]. Differential gene screening was performed according to the method of Huang et al. [50], and gene matching was calculated using fatuts v1.6.2 and the FPKM. An analysis of the differential expression of genes between the two groups was performed using DESeq2 v1.22.1, corrected *p*-values were calculated using the Benjamini–Hochberg method, and the threshold for significant differential expression was calculated using corrected *p*-values and the |log2foldchange|. Extensively targeted metabolomic analyses were performed using the method of Li et al. [51], based on the self-built Metware database, lyophilized samples were analyzed for absorbed extracts using an LC–electrospray ionization–MS/MS system(CNWBOND Carbon-GCB SPE Cartridge, 250 mg, 3 mL; Shanghai Ampere Scientific Instruments, Shanghai, China), and metabolites were quantified using a multiple reaction monitoring method. Metabolites with a variable importance in projection score > 1 and with a difference of >2 times or <0.5 times between the control and experimental groups were considered to be significantly different [30]. 

### 4.3. Physiological Indicators

Soluble protein, soluble sugar, proline, peroxidase, malondialdehyde, and chlorophyll levels were determined [52] using kits produced from the NanJing JianCheng Bioengineering Institute (Nanjing, China, http://www.njjcbio.com), according to the manufacturers’ instructions (Supplementary Table S1). Three biological replicates were performed for each physiological indicator, while three technical replicates were performed for each sample assayed.

### 4.4. Construction of Weighted Gene Co-Expression Network Analysis

After filtering low-expression genes, the WGCNA package (version 1.6.1) in the R (version 4.2.1) program was used to screen the genes (Supplementary Table S2). A total of 8000 genes were entered into the WGCNA to construct the gene co-expression network [53]. Then, pickSoftThreashold in the WGCNA package was used to calculate the weight values so that the network conformed to the scale-free network distribution. Ultimately, we chose β = 22 to construct the neighbor-joining matrix between genes and transformed the neighbor-joining matrix into a topological overlap matrix using the topological overlap matrix similarity algorithm to reduce noise and pseudo correlation. The default settings of the blockwiseModules function were used to construct the scale-free network. According to WGCNA, the genes were divided into 11 modules, which were correlated with the physiological indexes and metabolic data (trait indexes). Finally, we calculated the correlation between each module and the associated traits. 

### 4.5. Identification of Specific Modules and GO and KEGG Functional Enrichment Analysis

To identify the specific modules related to low-temperature stress, we calculated the correlation coefficients (r) and *p*-values of the module eigengene for each module with different traits. In this study, modules with |r| > 0.60 and *p* < 0.05 were selected as specific modules, and the resulting module genes were further subjected to GO and KEGG enrichment analyses using the clusterProfile package in the R program [54]. After correction by multiple hypothesis testing, genes with *p* < 0.05 were considered significantly enriched. 

### 4.6. Identification of Core Genes of Specific Modules and Construction of Gene Interaction Network

Gene connectivity within a module represents the regulatory relationship between genes and other genes; higher connectivity reflects the regulatory role of the gene in the module and indicates a greater likelihood of the gene being a potential hub gene. Therefore, by calculating the module KME values of genes in the module, we initially screened the top 20 genes as candidate core genes. Then, we imported the top 20 genes into Cytoscape 3.9.1 software and used the cytonca plug-in to calculate the betweenness values for core gene screening and gene interaction network construction [55], combined with structural gene promoter regions (2000 bp upstream) and cis-acting element information for gene network visualization [56]. 

### 4.7. Identification of Transcription Factors

TFs, which play an important role in regulating the response to low-temperature stress, are molecular switches that regulate the expression of various abiotic stress response genes [57]. In this study, the protein sequences of the identified core genes were submitted to plantTFDB (http://plntfdb.bio.uni-potsdam.de/v3.0/ (accessed on 25 April 2024)) to perform a BLAST search for predicting TFs and TF families [58]. The resulting TFs were submitted to NCBI (https://www.1ncbi.nlm.nih.gov/Structure/bwrpsb/bwrpsb.cgi (accessed on 27 April 2024)) for a functional analysis [59] to further understand the function of the core genes. The distribution of conserved motifs for the screened core genes was predicted using multiple expectation maximizations for motif excitation (MEME, http://meme-suite.org/tools/meme (accessed on 29 April 2024)) [60]. 

### 4.8. Real-Time Fluorescence Quantitative PCR Validation

To verify the reliability of gene expression, all samples of the six core genes were selected, and each sample was subjected to three technical replicates; in addition, three biological replicates were set up for each core gene to ensure the accuracy of the results, while RT-qPCR was performed afterwards to verify the results. The primers for the related genes used for the RT-qPCR analysis were designed in Beacon Designer 7.9., and ACT-1 gene was selected as the internal reference gene [61,62]. Then, RT-qPCR was performed using PerfectStart SYBR qPCR Supermix (TransGen Biotech, Beijing, China), the reaction volume was 20 µL (Table 4), and the thermal cycling conditions were set to 94 °C (30 s), 94 °C (5 s), 60 °C (30 s), and 40 cycles, and finally, the 2^−ΔΔCT^ method was used toto calculate the relative gene expression levels [63].

### 4.9. Statistical Analysis

Three biological replicates were performed for each experiment. The data obtained were analyzed for variance and correlation (Pearson’s correlation analysis) using SPSS (Ver. 23.0), and the results of the analysis of variance were expressed as mean ± standard deviation.

## 5. Conclusions

Quinoa seedlings respond to low-temperature stress by regulating osmotic adjustment substances such as proline, soluble sugar, and soluble protein and antioxidant enzyme activity. According to WGCNA, we identified two key gene modules and screened 12 core genes related to the response to low-temperature stress, 5 of which belonged to AP2/ERF, MYB-related, NAC, and C2C2-CO-like TF families. Among them, AP2/ERF was highly homologous to genes with known functions, suggesting that these candidate genes may play a key role in the response of quinoa seedlings to low-temperature stress. This study lays a foundation for further research on the molecular mechanism by which quinoa and other similar plants achieve resistance to low-temperature stress.

## Figures and Tables

**Table 1:** Correlation analysis of physiological traits in quinoa seedlings under low-temperature stress.

	Proline	Malondialdehyde	Soluble Sugar	Peroxidase	Soluble Protein	Total Chlorophyll
Proline	1					
Malondialdehyde	−0.519 *	1				
Soluble sugar	0.414	−0.059	1			
Peroxidase	−0.395	−0.022	0.037	1		
Soluble protein	−0.186	0.168	−0.095	−0.397	1	
Total chlorophyll	−0.199	−0.692 **	−0.088	−0.599 **	0.112	1

Note: * *p* < 0.05; ** *p* < 0.01; the positive number indicates positive correlation, and the negative number indicates negative correlation.

**Table 2:** Functional annotation of core genes in the specific module related to low-temperature stress resistance.

Module	Module Candidate Hub Genes	TFs Family	Gene Function
Turquoise	*gene-LOC110684437*	AP2/ERF	Ethylene-responsive transcription factor RAP2-12-like
	*gene-LOC110697250*	-	Pre-mRNA-processing factor 19-like
	*gene-LOC110709726*	-	14-3-3 protein 10-like
	*gene-LOC110726534*	-	Eukaryotic peptide chain release factor subunit 1-3
	*gene-LOC110722386*	-	Zinc finger CCCH domain-containing protein 40-like
	*gene-LOC110733109*	-	E3 ubiquitin-protein ligase Hakai-like
Gray	*gene-LOC110731664*	MYB-related	Protein LHY-like
	*gene-LOC110736639*	NAC	NAC domain-containing protein 2-like
	*gene-LOC110733919*	C2C2-CO-like	Zinc finger protein CONSTANS-LIKE 6-like
	*gene-LOC110683707*	-	40S ribosomal protein S23
	*gene-LOC110718876*	-	Uncharacterized LOC110718876
	*gene-LOC110720903*	AP2/ERF	Ethylene-responsive transcription factor 3-like

**Table 3:** Primer sequences to validate genes.

Quantity	Gene-ID	NCBI-Gene ID	Primer	5′ to 3′
1	*gene-LOC110722386*	110722386	Forward primer	AAGAGTGATGTCAATAGG
			Reverse primer	CCGTAAGAGGATTCATAA
2	*gene-LOC110733109*	110733109	Forward primer	GACTCAATTTGCTATCTATG
			Reverse primer	TCACTCATCTCTTCTCTT
3	*gene-LOC110733919*	110733919	Forward primer	AAGCAAGAGTATCAAGAT
			Reverse primer	TCAACTTCCTAACTTCATA
4	*gene-LOC110683707*	110683707	Forward primer	CTTACAAGAAGTCACATCT
			Reverse primer	AGTTCAAACAACCATCAT
5	*gene-LOC110718876*	110718876	Forward primer	GGATTGGATGAGAATAACT
			Reverse primer	GCTCTTAACCTCTGTAAC
6	*gene-LOC110720903*	110720903	Forward primer	TCTAATCACCACCATCAA
			Reverse primer	CTCAACCGTACTACTCAT
Internal reference gene	*ACT-1*	818339	Forward primer	GTCCACAGAAAGTGCTTCTAAG
			Reverse primer	AACAACTCCTCACCTTCTCATG

**Table 4:** Reaction system and conditions for qPCR 20 µL.

Component	Volume (µL)
2× PerfectStartTM SYBR qPCR Supermix	10
Passive Reference Dye (50×) (optional)	0.4
Nuclease-free Water	6.8
Forward Primer (10 μM)	0.4
Reverse Primer (10 μM)	0.4
cDNA (200 µg/µL)	2
Total volume	20

## Data Availability

All relevant data can be found within the manuscript and its Supplementary Materials. The datasets generated and analyzed during the current study are available from the corresponding author upon reasonable request.

## Supplementary Materials

The following supporting information can be downloaded at https://www.mdpi.com/article/10.3390/ijms25136885/s1.
