# Supplementary material for: Identifying Core Genes Related to Low-Temperature Stress Resistance in Quinoa Seedlings Based on WGCNA
Source: Int J Mol Sci. 2024 Jun 23;25(13):6885. doi: 10.3390/ijms25136885 (PMC11241592; doi:10.3390/ijms25136885)
Supplement: Supplementary file 1 [file ijms-25-06885-s001.zip › Figure S1.pdf]

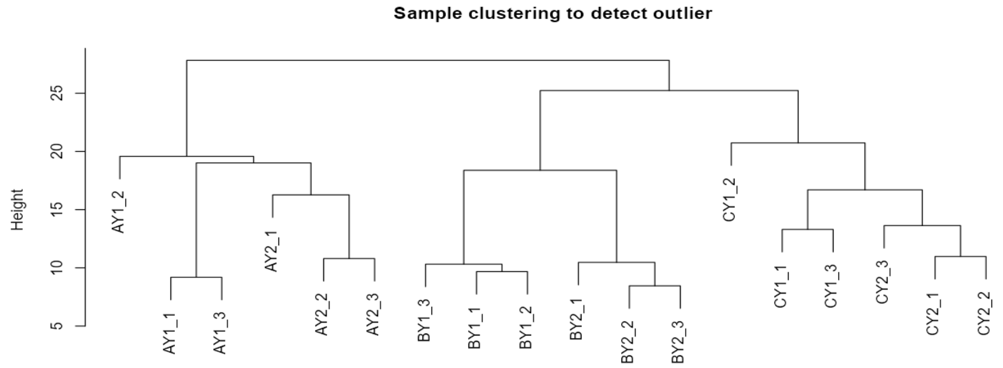

Figure S1. Clustering diagram of quinoa seedling samples. Horizontal coordinates represent quinoa seedling sample clustering, one column represents one sample, and clustering is based on gene expression similarity between quinoa seedling samples.
